# Anti-Amyloid Aggregation Activity of Black Sesame Pigment: Toward a Novel Alzheimer’s Disease Preventive Agent

**DOI:** 10.3390/molecules23030676

**Published:** 2018-03-16

**Authors:** Lucia Panzella, Thomas Eidenberger, Alessandra Napolitano

**Affiliations:** 1 Department of Chemical Sciences, University of Naples “Federico II”, Via Cintia 4, I-80126 Naples, Italy; alesnapo@unina.it; 2 School of Engineering and Environmental Sciences, Upper Austria University of Applied Sciences, Stelzhamerstraße 23, 4600 Wels, Austria; Thomas.Eidenberger@fh-wels.at

**Keywords:** black sesame, Alzheimer’s disease, vanillic acid, simulated digestion, acetylcholinesterase, butyrylcholinesterase, β-amyloid, β-secretase

## Abstract

Black sesame pigment (BSP) represents a low cost, easily accessible material of plant origin exhibiting marked antioxidant and heavy metal-binding properties with potential as a food supplement. We report herein the inhibitory properties of the potentially bioaccessible fraction of BSP following simulated gastrointestinal digestion against key enzymes involved in Alzheimer’s disease (AD). HPLC analysis indicated that BSP is transformed under the pH conditions mimicking the intestinal environment and the most abundant of the released compounds was identified as vanillic acid. More than 80% inhibition of acetylcholinesterase-induced aggregation of the β-amyloid Aβ1-40 was observed in the presence of the potentially bioaccessible fraction of BSP, which also efficiently inhibited self-induced Aβ1-42 aggregation and β-secretase (BACE-1) activity, even at high dilution. These properties open new perspectives toward the use of BSP as an ingredient of functional food or as a food supplement for the prevention of AD.

## 1. Introduction

Alzheimer’s disease (AD) is a neurodegenerative disorder that represents the most common type of dementia among elderly people. Thus far, low levels of acetylcholine (ACh), oxidative stress, disequilibrium of metals metabolism, and β-amyloid (Aβ) deposits have been considered to play definite roles in AD pathogenesis [1,2,3,4,5]. There is in particular a general consensus that Aβ is the main culprit of a straightforward cascade process that begins with its increased formation by processing of the amyloid precursor protein (APP) by β-secretase (BACE-1) and acetylcholinesterase (AChE)-induced or self-aggregation into oligomers and fibrils [6,7]. Synaptic dysfunction, tau protein hyperphosphorylation and aggregation, neuroinflammation, and oxidative stress would then follow, leading eventually to neuronal death and neurotransmitter deficits [8,9]. The first and so far sole marketed anti-Alzheimer drugs are the AChE inhibitors donepezil, rivastigmine, and galantamine, which increase the ACh levels [10]; these drugs, however, are effective only for symptomatic treatment of AD, since they are not able to prevent the progression of the disease. Other drugs, such as memantine, have proved to be effective in the treatment of both mild and moderate-to-severe AD by reducing excitotoxicity [11]. In the past years, there has been intense research activity for developing drugs able to inhibit Aβ formation or aggregation [12]. However, a number of clinically advanced Aβ-directed drug candidates have recently failed to show efficacy over placebo. This has been ascribed, at least in part, to the fact that AD might not result from a single process but from a robust network of interconnected events, whereby Aβ aggregation is just one of the causes. Therefore, therapies with combination of drugs [13] or the development of multitarget anti-Alzheimer drugs [14] has become a primary objective. These have been usually designed to hit at least Aβ formation and aggregation and AChE activity, especially prompted by the finding that AChE can bind Aβ and accelerate its aggregation through the so-called peripheral anionic site (PAS) [15,16]. The design of inhibitors able to simultaneously reach both the PAS and the catalytic anionic site (CAS) of AChE has therefore emerged as a promising source of multitarget anti-Alzheimer compounds [17,18,19,20,21].

Phenolic compounds have been the focus of increasing interest over the past decades because of a range of biological properties that have been implicated to account for the lower risk of malignant neoplasms and of cardiovascular and neurodegenerative diseases such as AD [22,23,24,25,26], although safety issues in the production of food supplements should not be disregarded [27,28]. In particular, sesame lignans, such as sesaminol and sesamol, have been shown to efficiently inhibit AChE and Aβ oligomerization and fibril formation [29,30,31,32]. Little attention has been directed so far to the potential AD prevention properties of the insoluble pigment of black sesame seeds. Black sesame (*Sesamum indicum* L.) seeds, traditionally used in Chinese folk medicine and as food for humans in China and other East Asian countries, have attracted interest because of their potent antioxidant activity, superior to that of white sesame seeds [33,34,35]. This activity has been commonly attributed to a number of soluble lipids of the lignan type, including primarily sesamin and sesamolin [36] but also sesaminol, sesamol, and pinoresinol [37,38], which occur in sesame seeds partly as glycosylated derivatives [39] and have been shown to display several health beneficial effects [40,41,42,43,44]. More recently, several studies have been focused on the peculiar properties of the insoluble pigment of black sesame seeds [45]. In particular, we recently developed an improved purification procedure to obtain black sesame pigment (BSP), involving fat removal by treatment of ground black sesame seeds with dichloromethane followed by an optimized hydrolytic protocol with 6 M HCl, at 100 °C, overnight [46]. The black pigment thus obtained displayed good antioxidant efficiency in the 2,2-diphenyl-1-picrylhydrazyl (DPPH) radical assay, good ferric ion-reducing capacity and potent antinitrosating properties. BSP was also shown to efficiently bind heavy metals like lead, cadmium, and mercury [47] that are commonly found in food and are known to cause impairment of the immune and central nervous systems [48,49]. 

The importance of evaluating the actual bioavailability of phytochemicals in food based on data concerning their absorption, metabolism, tissue and organ distribution, and excretion has been increasingly appreciated [50,51,52]. Such studies carried out on animals or human subjects are necessarily complex, expensive, and lengthy. This is the reason why different in vitro procedures that mimic the physiochemical and biochemical conditions encountered in the gastrointestinal tract have been developed, providing preliminary data on the potential bioavailability of different components of the food under evaluation [53,54,55,56,57]. 

We report herein the results of chemical assays to evaluate the potential AD preventive effect of the bioavailable fraction of BSP following a simulated gastrointestinal digestion. The method consisted of two sequential steps, that is an initial pepsin/HCl digestion, at pH 1.7, for 2 h at 37 °C, followed by a digestion with bile salts/pancreatin for 2 h at 37 °C at pH 7.5, to simulate pH and transit times in the stomach and small intestine, respectively. 

## 2. Results and Discussion

### 2.1. Structural Investigation of BSP Phenolic Components

**Figure 1:**
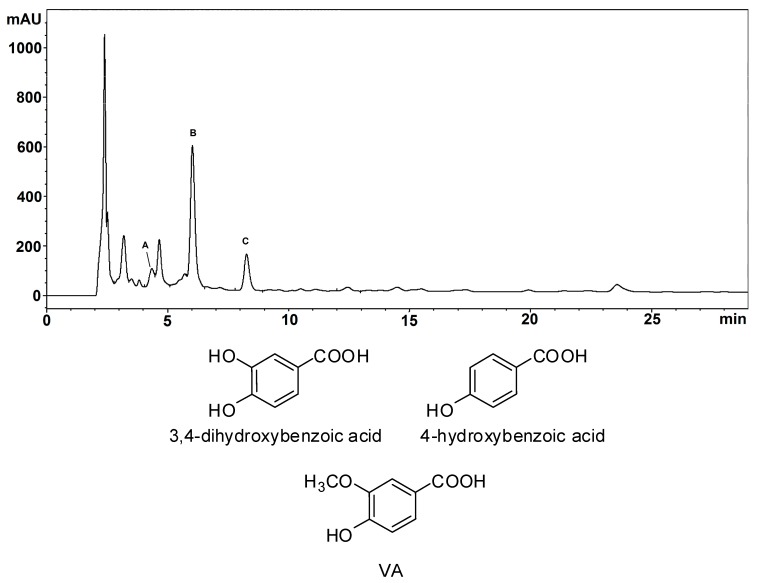
HPLC trace (detection at 254 nm) of the ethyl acetate extractable fraction of the alkali fusion mixture of BSP. Peak A: 3,4-dihydroxybenzoic acid; peak B: 4-hydroxybenzoic acid; peak C: VA. The chromatographic separation was performed on an octadecylsilane (ODS) column (325 × 4.60 mm, 5 µm), at a flow rate of 0.9 mL/min. The mobile phase was a 0.02 M ammonium acetate buffer, pH = 3.5 (solvent A)/methanol (solvent B) gradient as follows: from 5% to 25% B, 0–20 min. Chemical structures of the identified compounds are also shown.

Based on the previous finding that vanillic acid (VA) represents one of the main fragments following chemical degradation of BSP [46] and on the results of total reflectance Fourier transform infrared (ATR-FTIR) and solid-phase NMR experiments [47], it has been proposed that *ortho*-methoxyhydroxy units are represented to a significant extent in the backbone of BSP, although *ortho*-diphenols and phenolic acids are present as well. To further confirm this hypothesis, BSP was subjected to alkali fusion under reducing conditions. HPLC/DAD/MS analysis of the ethyl acetate extractable fraction indicated the presence of five main products, three of which were identified by comparison with authentic reference standards as 3,4-dihydroxybenzoic acid, 4-hydroxybenzoic acid and VA (Figure 1, Figures S1 and S2). These results confirm the presence of phenol, *ortho*-diphenol, and *ortho*-methoxyphenol moieties in BSP.

### 2.2. Simulated Gastrointestinal Digestion of BSP

**Figure 2:**
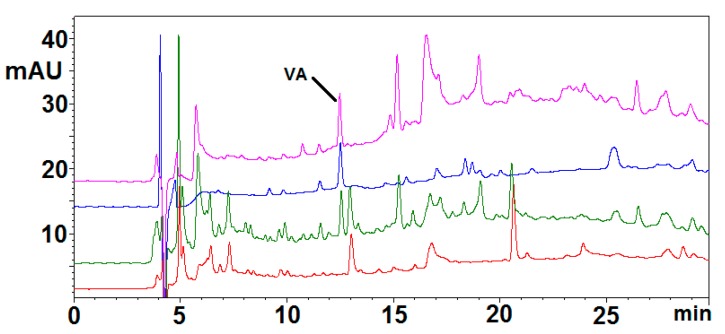
HPLC traces of the IN samples obtained from simulated gastrointestinal digestion of BSP. Green: IN sample from BSP digestion. Blue: ethyl acetate extractable fraction of the IN sample. Red: IN sample from a control mixture containing only enzyme and bile salts (without BSP). Purple: IN sample from BSP digestion without enzyme and bile salts. The chromatographic separation was performed on a ODS column (250 × 4.60 mm, 5 µm), at a flow rate of 0.7 mL/min. The mobile phase was a 1% formic acid (solvent A)/methanol (solvent B) gradient as follows: from 5% to 90% B, 0–45 min.

Among the different protocols reported in the recent literature mimicking the physiochemical and biochemical conditions encountered in the gastrointestinal tract, a procedure was chosen that had been used to investigate release of phenolic compounds from food matrix [58,59]. A suspension of the pigment at 5 mg/mL was subjected to two sequential steps: an initial pepsin/HCl digestion for 2 h at 37 °C to simulate gastric conditions, followed by a digestion with bile salts/pancreatin for 2 h at 37 °C at pH 7.5 to simulate small intestine conditions. An oral digestion step using α-amylase was not included in the digestion protocol as the acid treatment used to obtain BSP likely produces efficient removal of the carbohydrate components [47]. In order to assess the potential serum availability of the components released from the pigment after complete digestion, a model was used consisting of a cellulose dialysis tube (molecular mass cutoff 12 KDa), containing sufficient sodium bicarbonate to neutralize the acidity of the mixture after the initial pepsin digestion, immersed into the mixture containing the pigment at the time the bile salts/pancreatin digestion treatment starts. The bicarbonate is slowly released into the mixture and some of the components are allowed to enter the tube. After 2 h at 37 °C the solution outside the dialysis tubing was taken as the OUT sample, representing the material remaining in the gastrointestinal tract, and the solution that entered the dialysis tubing was taken as the IN sample representing the material entering the serum. Care should be taken, however, in extrapolating the results to the in vivo situation because the partition of phenols into the dialysis tubing is dependent also on their diffusion rates and stability. HPLC analysis of the IN sample (Figure 2, green trace) indicated a complex pattern of products, some of which were observed also in a control mixture not containing BSP and therefore clearly due to enzyme/bile salts-derived products (Figure 2, red trace). The remaining components were also present in the mixture of the pigment exposed to the pH conditions of the simulated digestion treatment but without added enzymes and bile salts (Figure 2, purple trace), indicating that BSP is able to release low molecular weight compounds under the conditions of the intestinal transit without the need of enzyme action. Of these compounds, only that eluted at 12.5 min was quantitatively extracted in ethyl acetate (Figure 2, blue trace). No appreciable release of any of these compounds could be observed at the acidic pH simulating gastric conditions (not shown).

LC/ESI+/MS analysis allowed to identify the product eluted at 12.5 min as VA (pseudomolecular ion [M + H]^+^ at *m*/*z* 169), confirmed also by comparison of the chromatographic behavior with that of an authentic reference standard (Figure S3). This result is of considerable interest in the light of the several health beneficial properties of VA [60,61,62,63,64], first of all its neuroprotective activity [65,66]. The concentration of VA released in the IN sample was estimated to be as high as 6 μM. However, it should be considered that liver metabolism and the blood-brain barrier could potentially restrict the concentration of VA available in brain.

### 2.3. Determination of the Activity of BSP toward AD Drug Targets

The activity of the IN sample and, for comparison, of VA toward AD drug targets was evaluated by a number of validated chemical assays. Based on the results described above, to avoid interference by digestive enzymes and bile salts, the IN sample from BSP that has only been subjected to variations of pH was tested.

#### 2.3.1. AChE and BChE Inhibition Assay

The cholinergic hypothesis for AD suggests that cognitive deterioration is mainly caused by low levels of acetylcholine (ACh) because maintaining high ACh levels (via AChE inhibitors) has been shown to alleviate these symptoms [67]. The AChE inhibition properties of VA were evaluated by the Ellman’s method, using AChE from electricus eel and acetylthiocholine iodide as substrate, as described in several papers [68,69,70]. Indeed, it was not possible to test the IN sample since it exhibited a significant absorption at the detection wavelength used in the Ellman’s method (412 nm) even at high dilution, therefore leading to interference. The percentage of inhibition as a function of log-scale concentration of VA is reported in Figure S4. More than 45% inhibition was observed with 180 μM VA, whereas a ca. 15% inhibition was observed at 6 μM, that is at the concentration released from BSP upon simulated gastrointestinal digestion.

BChE is another target of interest in the search for anti-Alzheimer drugs as this enzyme plays a critical role for ACh hydrolysis in the late stage of AD, when level of AChE in brain declines to 55–67% of normal values while BuChE increases to 120% of normal levels [71,72]. The inhibition activity of VA was evaluated as described above for AChE, using equine serum BChE and butyrylthiocholine iodide as the substrate. As shown in Figure S5, a maximum 27% inhibition was observed with 18 μM VA, but a comparable inhibition (18%) as that determined in the AChE inhibition assay was observed at the potentially physiological concentration of 6 μM.

#### 2.3.2. AChE-Induced Aβ1-40 Aggregation Inhibition Assay

The inhibition properties of VA and the IN sample against AChE-induced amyloid aggregation were evaluated using the thioflavin T-based fluorescent method [69] and the amyloid Aβ1-40, which represent one of the main isoforms of Aβ peptides [73,74]. A 83 ± 5% inhibition was observed with 5 μM VA, and a comparable value (83 ± 7%) was obtained with a 20-fold diluted IN sample. These results are very encouraging in the light of the low, potentially physiological, concentrations tested, especially for the IN sample. Based on the concentration determined by HPLC analysis for VA in the IN sample (ca. 6 μM), these results would suggest that other components significantly contribute to the aggregation inhibition activity. The characterization of these compounds is currently underway.

Notably, the inhibition induced by BSP-related samples was higher than that reported for donepezil in a human erythrocytes AChE-induced Aβ1-40 aggregation assay (25.0 ± 0.6% at 100 μM) [75]. 

Compared with the results of the AChE inhibition assay (only 15% inhibition at 5 μM), these results would indicate a higher affinity of VA for PAS than for CAS of AChE.

#### 2.3.3. β-Amyloid Self-Aggregation Inhibition

**Figure 3:**
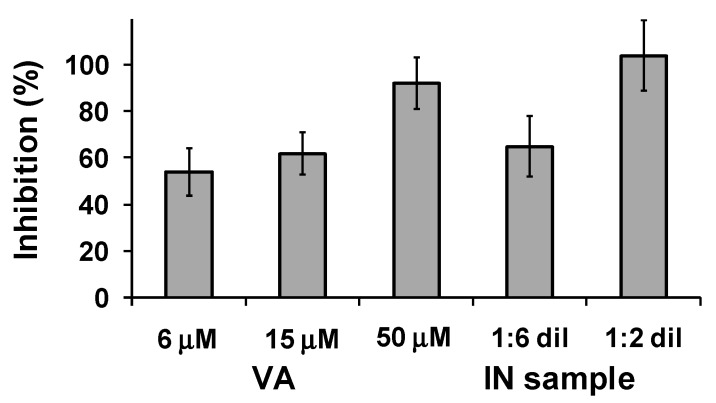
Inhibition of self-induced Aβ1-42 aggregation by VA and IN sample at different dilutions. Reported are the mean ± SD values from at least three experiments.

The inhibitory activity of the samples against the spontaneous aggregation of amyloid Aβ1-42, which shows a significant increase with certain forms of AD [74,76], was determined using the thioflavin T based fluorometric assay as before. In this case a ca. 50% inhibition was observed with 6 μM VA, reaching a ca. 90% value at 50 μM. An even higher inhibition was observed with the IN sample (Figure 3). Under the same conditions, it was reported that 25 μM donepezil did not show any inhibitory activity, whereas curcumin at 25 μM showed 43% inhibition [75]. 

Copper-induced β-amyloid aggregation has also been implicated in the etiology of AD [77]. The ability to inhibit copper-induced amyloid aggregation is related to the capacity of a compound to form copper chelates in order to retrieve the metal ion from the amyloid. This is not the case of VA, which did not form complexes with copper ions [78], so the possibility that VA could interfere with the copper-induced amyloid aggregation was not investigated because it is rather unlikely.

#### 2.3.4. BACE-1 Inhibition

**Figure 4:**
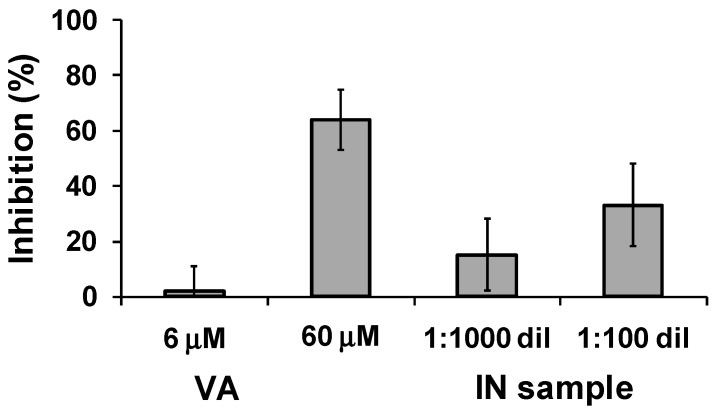
Inhibition of BACE-1 by VA and IN sample. Reported are the mean ± SD values from at least three experiments.

BACE-1 catalyzes the first and rate-limiting step of the biosynthesis of β-amyloid peptide from APP, thereby constituting a primary target in the search for anti-AD drugs [79,80]. The BACE-1 inhibition assay was performed by employing a model peptide mimicking the APP sequence as substrate and the fluorescence resonance energy transfer (FRET) method [81]. An inhibition higher than 60% was caused by 60 μM VA, but essentially no effect was observed at the potentially physiological concentration of 6 μM. On the other hand, significant inhibition properties (15–33%) were found for the IN sample even at the high dilution used to eliminate any interference in the fluorescence measurement (Figure 4). As an example, previously reported IC_50_ values for known inhibitors of BACE-1 used as positive controls in BACE-1 FRET assay kits were ca. 12 μM for morin [81], 7.4 μM for quercetin [82], and 33 nM for the peptidomimetic inhibitor OM99-2 [83].

## 3. Materials and Methods

### 3.1. Materials

BSP was prepared from black sesame seeds (*Sesamum indicum* L.) as previously described [46]. Pepsin from porcine gastric mucosa, pancreatin from porcine pancreas, porcine bile extract, 4-hydroxy-3-methoxybenzoic acid (VA), 4-hydroxybenzoic acid, 3,4-dihydroxybenzoic acid, KOH, NaOH, Na_2_S_2_O_4_, AChE from *Electrophorus electricus* (E.C.3.1.1.7, Type VI-S), acethylthiocholine iodide, 5,5′-dithiobis-2-nitrobenzoic acid (DTNB), BChE from equine serum (E.C.3.1.1.8), butyrylthiocholine iodide, thioflavin T, 1,1,1,3,3,3-hexafluoro-2-propanole (HFIP) and BACE1 activity detection kit were purchased from Sigma-Aldrich (Milan, Italy); synthetic Aβ 1-40 and 1-42 (human sequence) were from Bachem (Bubendorf, Switzerland).

### 3.2. General Experimental Methods

HPLC analysis of the alkali fusion mixtures was performed with an instrument equipped with a diode array detector (DAD). The chromatographic separation was performed on an octadecylsilane (ODS) column (325 × 4.60 mm, 5 µm), at a flow rate of 0.9 mL/min. The mobile phase was a 0.02 M ammonium acetate buffer, pH = 3.5 (solvent A)/methanol (solvent B) gradient as follows: from 5% to 25% B, 0–20 min. The principle detection wavelength was 254 nm. UV-VIS spectra were collected for all peaks in the range 200–600 nm. LC-MS analysis was performed with a system equipped with a quadrupole mass spectrometer with ESI source operating in negative ionization mode in the following conditions: nebulizer pressure 50 psi; drying gas (nitrogen) 11 L/min, 300 °C; capillary voltage 4500 V; fragmentor voltage 150 V. The chromatographic separation was performed as described above for HPLC analysis. 

HPLC analysis of the simulated gastrointestinal digestion mixtures was performed with an instrument equipped with a UV-vis detector. The chromatographic separation was performed on a ODS column (250 × 4.60 mm, 5 µm), at a flow rate of 0.7 mL/min. The mobile phase was a 1% formic acid (solvent A)/methanol (solvent B) gradient as follows: from 5 to 90% B, 0–45 min. The detection wavelength was 254 nm. LC-MS analysis was performed with a system equipped with a UV-vis detector and a quadrupole mass spectrometer with ESI source operating in positive ionization mode in the following conditions: nebulizer pressure 50 psi; drying gas (nitrogen) 10 L/min, 350 °C; capillary voltage 4000 V; fragmentor voltage 50 V. The chromatographic separation was performed as described above for HPLC analysis.

### 3.3. Alkali Fusion of BSP

Solid and finely grinded KOH, NaOH, and Na_2_S_2_O4 were mixed in a 1:1:0.02 ratio (i.e., 100 mg/100 mg/2 mg) in a pyrex tube and kept at 240 °C for 3 min (until fusion of the reagents). 20 mg of BSP were then added and the mixture was kept at 240 °C for 10 min. After cooling to room temperature, 10 mL of a 1% sodium dithionite solution were added. After addition of 0.7 mL of acetic acid, the mixture was extracted with ethyl acetate (3 × 15 mL) and the combined organic layers were dried over sodium sulfate and evaporated to dryness. The solid residue was reconstituted in mobile phase, cleared by filtration (0.2 µm syringe filters), and analyzed by HPLC/DAD/MS. 

### 3.4. Simulated Gastrointestinal Digestion of BSP

The procedure was adapted from a reported method [58,59]. BSP (100 mg) was finely suspended by use of a glass/glass Tenbroeck grinder in 2.5 mL of distilled water. The suspension was made up to 20 mL with distilled water (final concentration of BSP 5 mg/mL) and adjusted to pH 1.7 with 5 M HCl. 315 Units/mL pepsin were added and the mixture was incubated at 37 °C under stirring in the dark. After 2 h, 20 mg of pancreatin and 125 mg of bile extract dissolved in 5 mL of 0.1 M NaHCO_3_ were added, followed by a segment of cellulose dialysis tubing (molecular mass cutoff 12 kDa) containing 1.8 mL of 1 M NaHCO_3_. After 2 h of incubation at 37 °C, the solution that entered the dialysis tubing (taken as the IN sample) was separated, analyzed by HPLC and LC/MS and stored at −20 °C. In other experiments, BSP was exposed to the pH conditions of the general procedure, but without addition of the enzyme components and bile salts and the deriving IN sample was tested for its potential activity toward AD drug targets. When required, the IN sample was taken to pH 3 and extracted with ethyl acetate. Control experiments were run without addition of BSP. 

### 3.5. AChE and BChE Inhibition Assays

The assays were performed as described [69] with slight modifications. Briefly, to 1.2 mL of 0.1 M phosphate buffer (pH 8.0) 40 μL of a solution of VA at different concentrations (0.006–6 mM) were added, followed by 40 μL of a 0.01 M ethanolic solution of DTNB and 40 μL of a 2.5 U/mL aqueous solution of the enzyme. After 5 min 8 μL of a 0.075 M aqueous solution of the substrate was added and after additional 2.5 min the absorbance at 412 nm was measured. For the reference value, 40 μL of water replaced the VA solution. For determining the blank value, 40 μL of water replaced the enzyme solution.

### 3.6. AChE-Induced Aβ1-40 Aggregation Inhibition Assay

The assay was performed as described [69] with slight modifications. The Aβ1-40 solution was prepared by suspending 1 mg of amyloid in 50 μL of HFIP; the suspension was left to stay at room temperature for 4 h, after that the solvent was removed under a stream of argon. Briefly, to 0.15 mL of buffer solution (25 mM NaH_2_PO_4_, 100 mM NaCl, pH 8.0) 15 μL of 100 μM solution of VA or of IN sample, followed by 120 μL of a 2.5 U/mL buffer solution of AChE and 15 μL of a 2 mM DMSO solution of HFIP-pretreated Aβ1-40 were added. After 5 h 60 μL of a 15 μM buffer solution of thioflavin T were added and after additional 5 min the fluorescence emission at 482 nm (λex = 450 nm) was measured. For the reference value, 15 μL of water replaced the inhibitor solution. For determining the blank value, 15 μL of DMSO replaced the Aβ solution.

### 3.7. Self-Induced Aβ1-42 Aggregation Inhibition Assay

The assay was performed as described [75] with slight modifications. A 0.5 mM stock solution of Aβ1-42 was prepared in DMSO and diluted 1:10 in 0.05 M phosphate buffer solution containing 0.1 M NaCl (pH 7.5). To 30 μL of the amyloid solution 30 μL of 10–100 μM solution of VA or of IN sample (undiluted or diluted 1:3 in water) were added. After 24 h incubation at 37 °C, 240 μL of a 5 μM thioflavin T in 50 mM glycine-NaOH buffer (pH 8.5) were added and after 5 min the fluorescence emission at 490 nm (λex = 446 nm) was measured. For the reference value, 30 μL of water replaced the inhibitor solution.

### 3.8. BACE-1 Inhibition Assay

The assay was carried out according to the supplied manual. Briefly, to 1.02 mL of buffer 150 μL of 60–600 μM VA solution or of IN sample (diluted 1:10 or 1:100 in water) were added, followed by 300 μL of the substrate solution and 30 μL of enzyme solution. After 2 h incubation at 37 °C, 600 μL of the stop solution were added and the fluorescence emission at 405 nm (λex = 320 nm) was measured. For the reference value, 150 μL of water replaced the inhibitor solution. For determining the blank value, 30 μL of buffer replaced the enzyme solution.

## 4. Conclusions

We have reported herein that black sesame pigment is transformed under the conditions of simulated gastrointestinal digestion, although the amount of the low molecular weight components released is relatively low (<10% on a *w*/*w* basis) compared to the pigment treated. Most of the transformations occurred under the pH conditions mimicking the intestinal environment, and were due to moderate alkaline hydrolytic reaction rather than the action of the enzymes.

All of the components released have a potential to pass into the serum as evidenced by the serum availability model used. One of the most abundant of these components proved to be vanillic acid, a result of considerable interest in light of the several beneficial properties of this compound.

More than 80% inhibition of the acetylcholinesterase-induced Aβ1-40 aggregation was observed in the presence of the potentially bioaccessible (soluble) fraction from the simulated digestion of the pigment, which also efficiently inhibited self-induced Aβ1-42 aggregation and β-secretase activity even after extensive dilution.

These properties open new perspectives toward the use of black sesame pigment, an easy to handle and stable material that could be obtained from a low cost source and purified through a scalable procedure, as a food supplement for the prevention of amyloid aggregation.

## Supplementary Materials

Supplementary materials are available online. Figure S1: HPLC trace of a standard mixture containing 3,4-dihydroxybenozic acid, 4-hydroxybenzoic acid and VA (same elution conditions of Figure 1), Figure S2: UV and mass spectra of the products A, B and C of Figure 1, Figure S3: MS spectrum of the product eluted at 12.5 min and HPLC trace of standard VA (under the same elution conditions of Figure 2), Figure S4: AChE inhibition by VA. Reported are the mean + SD values from at least three experiments, Figure S5: BChE inhibition by VA. Reported are the mean + SD values from at least three experiments.
