# Supplementary material for: Anti-Amyloid Aggregation Activity of Black Sesame Pigment: Toward a Novel Alzheimer’s Disease Preventive Agent
Source: Molecules. 2018 Mar 16;23(3):676. doi: 10.3390/molecules23030676 (PMC6017763; doi:10.3390/molecules23030676)
Supplement: Supplementary file 1 [file molecules-23-00676-s001.pdf]

Supplementary Materials

Anti-amyloid aggregation activity of black sesame pigment: toward a novel Alzheimer's disease preventive agent

Lucia Panzella [1,*], Thomas Eidenberger [2], and Alessandra Napolitano [1]

[1] Department of Chemical Sciences, University of Naples "Federico II", Via Cintia 4, I-80126 Naples, Italy; alesnapo@unina.it

[2] School of Engineering and Environmental Sciences, Upper Austria University of Applied Sciences, Stelzhamerstraße 23, 4600 Wels, Austria; Thomas.Eidenberger@fh-wels.at

* Correspondence: panzella@unina.it; Tel.: +39-081-674131

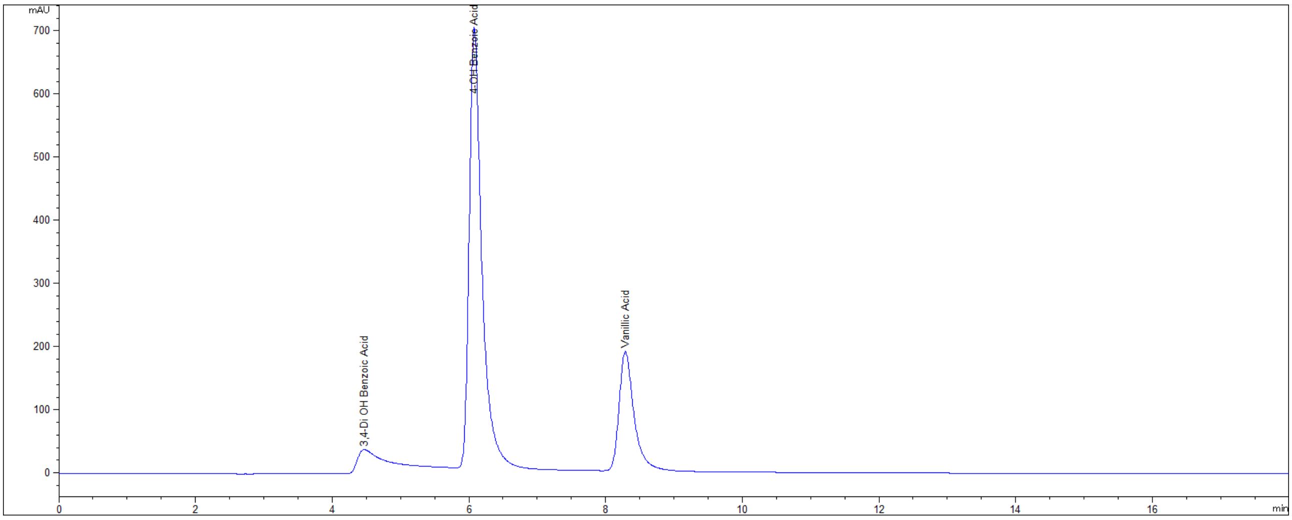

Figure S1. HPLC trace of a standard mixture containing 3,4-dihydroxybenozic acid, 4-hydroxybenzoic acid and VA (same elution conditions of Figure 1).

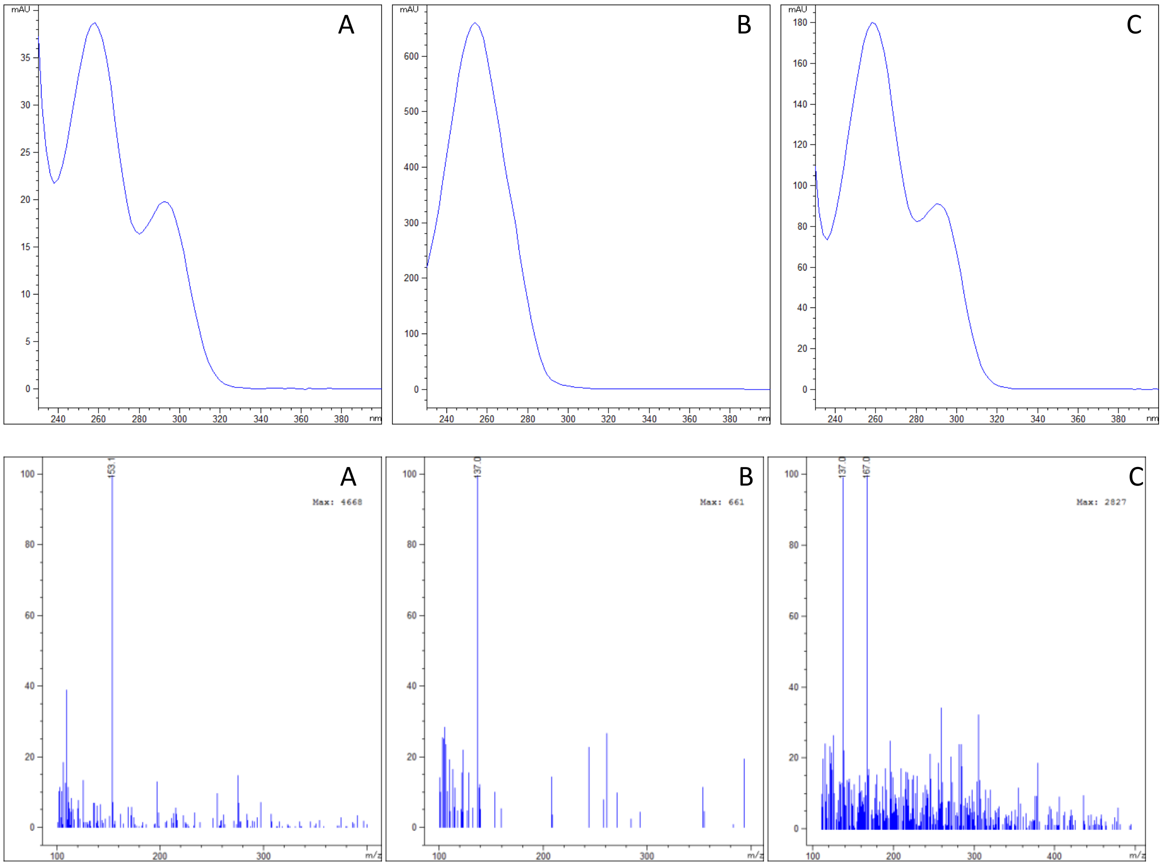

Figure S2. UV and mass spectra of the products A, B and C of Figure 1.

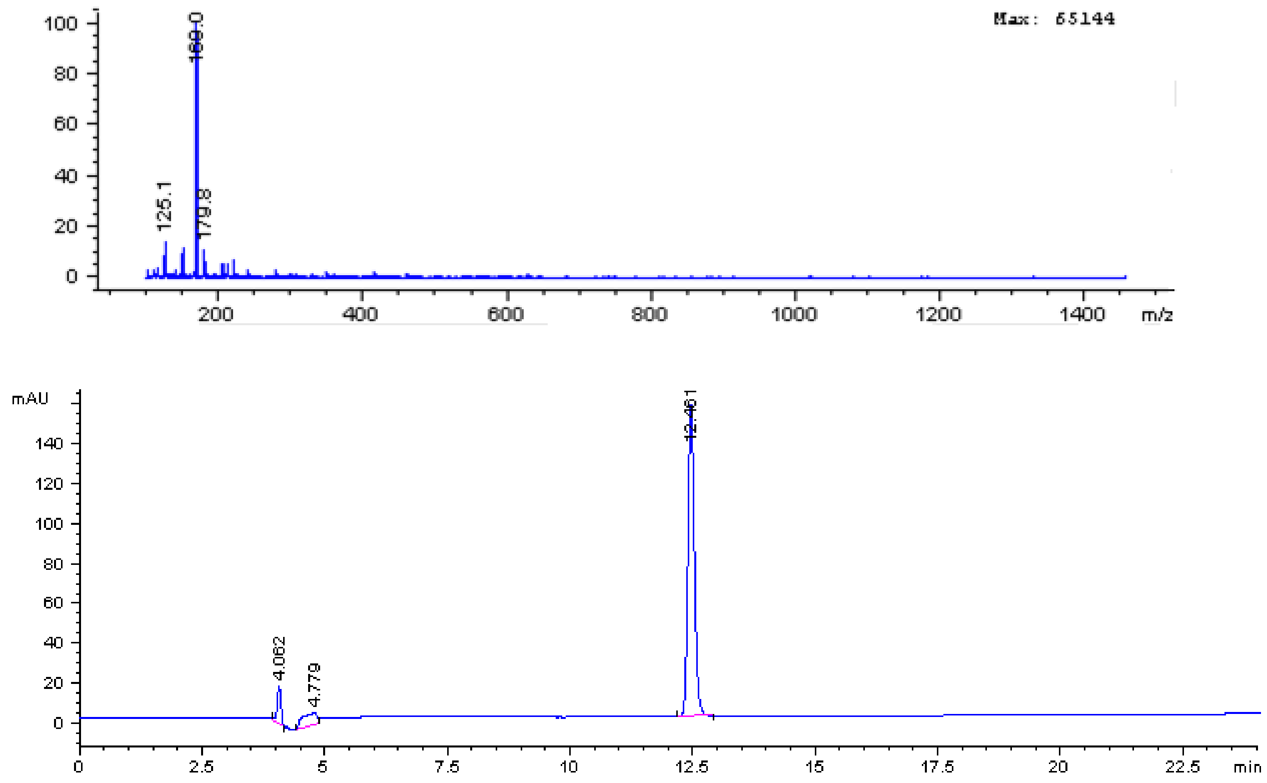

Figure S3. MS spectrum of the product eluted at 12.5 min and HPLC trace of standard VA (under the same elution conditions of Figure 2).

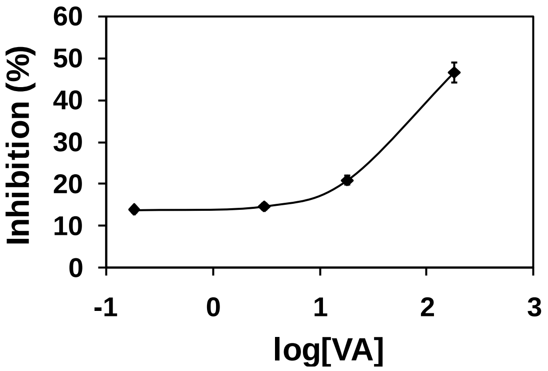

Figure S4. AChE inhibition by VA. Reported are the mean $\underline{+}$ SD values from at least three experiments.

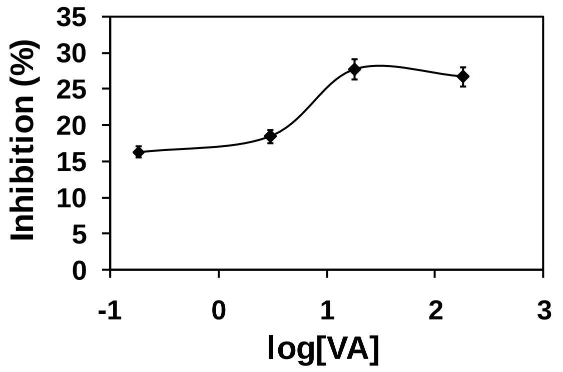

Figure S5. BChE inhibition by VA. Reported are the mean ± SD values from at least three experiments.
